# A hypergraph convolution-based intelligent healthcare platform for aging population management

**DOI:** 10.3389/fpubh.2025.1640808

**Published:** 2025-08-08

**Authors:** Wenjie Li, Bing Hou, Xiao-xiao Wang

**Affiliations:** ^1^The Academy of Social Sciences on Water Research in the New Era, North China University of Water Resources and Electric Power, Zhengzhou, China; ^2^School of Public Administration, North China University of Water Resources and Electric Power, Zhengzhou, China; ^3^School of Management, Henan University of Chinese Medicine, Zhengzhou Henan, China

**Keywords:** population aging, intelligent healthcare, integrated medical and aged care services, internet of thing, hypergraph convolutional network

## Abstract

**Introduction:**

The growing aging population imposes increasing demands on healthcare systems, particularly in managing chronic diseases among older adults. However, existing approaches face significant challenges in integrating multimodal data and analyzing complex disease associations effectively.

**Methods:**

This study proposes an intelligent healthcare platform based on Hypergraph Convolutional Networks (HGCN) to address these limitations. The platform collects real-time multimodal data—including physiological signals, behavioral records, and environmental parameters—via wearable and IoT devices. These data are integrated into a dynamic medical knowledge graph, and analyzed using HGCN and hierarchical feature learning to facilitate health condition monitoring and inter-institutional collaboration.

**Results:**

Experimental evaluations demonstrated the platform’s effectiveness, achieving an 87.26% accuracy and a 0.831 F1-score in disease risk prediction. The system also maintained a 100% request success rate under 480 concurrent users, with minimal response latency.

**Discussion:**

The proposed platform significantly improves personalized care for older adults, enhances the efficiency of healthcare resource allocation, and offers a scalable solution for intelligent healthcare services.

## 1 Introduction

With the rapid acceleration of global population aging, healthcare systems face unprecedented challenges due to the rising prevalence of chronic diseases, comorbidities, and the unique healthcare needs of older adults. These challenges are exacerbated by limited resources and delayed responses in traditional systems. According to the United Nations World Population Prospects 2023, by 2050, the proportion of the global population aged 65 years or older adults will reach 16%, with China’s older adults population expected to exceed 400 million, accounting for more than 30% of the total population (1). This demographic transformation has significantly escalated the burden of chronic illnesses. Data from the World Health Organization (WHO) reveals that 75% of older adults are afflicted with at least one chronic condition—such as hypertension, diabetes, or cardiovascular disease—placing immense pressure on healthcare infrastructures. Additionally, healthcare spending on older adult’s accounts for over 70% of total health expenditures, a statistic that underscores the critical need for more efficient and effective healthcare frameworks.

The conventional healthcare model, reliant on offline diagnosis, treatment, and centralized data management, is ill-equipped to address the modern demands of real-time monitoring, personalized interventions, and dynamic resource allocation (2). These shortcomings manifest as overstretched medical resources, inefficient service delivery, and outdated data management practices, all of which highlight the pressing need for innovative solutions (3). The integration of multimodal data, such as physiological signals (4), behavioral patterns (5), and environmental factors (6), is critical for addressing these challenges, but traditional systems struggle to model complex, higher-order relationships among these data types. This limitation hampers the ability to derive comprehensive insights from diverse data sources, further necessitating advanced approaches to healthcare delivery.

In response to these challenges, the integrated medical and aged care services (IMACS) model has emerged as a promising strategy, aiming to unify medical resources and aged care services through cross-sector collaboration (7). IMACS aims to consolidate medical resources and aged care services through cross-sector collaboration between healthcare and senior care institutions, thereby providing older adults with comprehensive and continuous health management and daily living support (8). However, current integrated medical and aged care services implementations face multiple systemic barriers, including homogeneous service models, uneven resource allocation, and pervasive data silos, which collectively hinder the fulfillment of diversified and personalized needs among aging populations. Consequently, enhancing the intelligent capabilities of integrated medical and aged care services through advanced information technologies has become an urgent priority for sustainable older adults care innovation.

In recent years, the rapid development of emerging technologies such as the IoT, big data, and artificial intelligence has provided new opportunities for the optimization of healthcare systems (9). In particular, techniques such as graph convolutional network (GCN) and HGCNs have shown great potential in multimodal data fusion and complex relationship modeling (10). Hypergraph-based methods, in particular, offer significant advantages over traditional GCNs by capturing higher-order relationships across multiple data modalities, enabling more accurate disease prediction and health management (11). Multimodal data fusion can integrate data from different sources (e.g., physiological indicators, behavioral logs, environmental parameters) and provide richer information for the comprehensive assessment of the health status of the older adults. And the HGCN, through its powerful higher-order relationship modeling capability, can more effectively mine the complex associations between data and provide more accurate support for disease prediction, risk assessment and personalized intervention (12).

Hypergraph neural network can effectively represent higher-order relationships between complex data by constructing hyper edges to connect multiple nodes, which has a unique advantage in multimodal information fusion in healthcare. It can integrate heterogeneous data such as physiological indicators, medical images, electronic medical records, etc., and improve the accuracy of disease diagnosis, risk prediction and health management (13). Cao et al. (14) proposed HGCN based on hypergraph spectral domain convolution. They introduced the hypergraph structure into the higher-order relationship modeling of medical data, and significantly improved the expressive power of disease association analysis by connecting multimodal nodes (e.g., patients, drugs, and symptoms) through hyper edges. Wu et al. (15) proposed a multimodal fusion method based on hypergraph convolution combined with underlying feature learning, and verified the effectiveness of the method in object recognition and structural body health monitoring. Sun et al. (16) proposed the Federated Hypergraph Learning (FedHG) method, which effectively achieves privacy-preserving collaborative analysis of cross-hospital electronic medical records through distributed hypergraph embedding aggregation techniques, and at the same time meets GDPR compliance requirements and demonstrates good performance in diabetes complication prediction. Lu et al. (17) designed a dynamic spatio-temporal hypergraph convolutional networks (ST-HGCN) model, which utilizes time-series hyperedges to model the evolutionary path of chronic diseases, such as the process from hypertension to heart failure to renal function abnormality, and thus has a significant accuracy improvement. Kumar et al. (18) introduced a hypergraph neural network with attention-based fusion for integrating multimodal medical data. Their approach effectively combines diverse data types, such as physiological signals, medical images, and clinical records, demonstrating improved performance in disease diagnosis and health management tasks. Ahmed et al. (19) proposed a hypergraph attention-based federated learning method for mental health detection. By leveraging hypergraph structures in a federated setting, their model enables privacy-preserving analysis of distributed mental health data while capturing complex relationships between multimodal features. They realized the alignment of physiological signals (e.g., ECG), imaging data (e.g., MRI) and textual medical records with the help of a cross-modal attention mechanism, and achieved a significant accuracy breakthrough in the early diagnosis of Alzheimer’s disease.

While existing studies demonstrate the value of HGCNs in healthcare, key gaps remain unaddressed. Cao et al. (14) focused primarily on static hypergraph representations for disease association analysis, lacking mechanisms to model temporal health deterioration (e.g., chronic disease progression). Ahmed et al. (19) advanced privacy-preserving federated learning but did not resolve over smoothing in multimodal feature fusion—a critical limitation for personalized older adults care where low-level physiological details (e.g., ECG anomalies) must be preserved. Unlike GCNs, which are limited to pairwise interactions, HGCNs can capture interactions among multiple entities (e.g., physiological signals, behavioural patterns, and environmental factors) through hyperedges, enabling a more comprehensive representation of the data. This is crucial in healthcare scenarios where health outcomes often depend on the interplay of multiple factors. Additionally, HGCNs facilitate effective multimodal fusion by integrating diverse data types into a unified hypergraph structure, which standard DL models struggle to achieve without extensive feature engineering.

This study aims to develop an intelligent healthcare management framework based on HGCNs to capture complex relationships across multimodal health data, including physiological signals, behavioral patterns, and environmental factors. It further introduces an adaptive feature learning mechanism that integrates both high-level and low-level features to enhance disease classification accuracy and mitigate oversmoothing. The proposed framework is implemented as a full-stack platform, supporting real-time monitoring, early warning, and reliable service delivery for older adults health management, validated through experiments on both benchmark and real-world datasets.

The key innovations of this study are as follows:

(1) Applies hypergraph convolutional networks (HGCNs) to a real-world older adults healthcare management scenario, modeling multimodal relationships such as physiological signals, behavioral patterns, and environmental conditions.
(2) Introduces a novel adaptive mechanism that combines high-level and low-level feature learning, preventing oversmoothing and improving classification performance in multimodal settings.
(3) By leveraging KNN-based hypergraph construction, the framework captures higher-order interactions across different health data types, going beyond traditional graph-based techniques that only model pairwise relationships.

The remainder of this paper is organized as follows: Section 2 details the methodology, beginning with the platform architecture in Section 2.1, which describes the overall design of the intelligent healthcare management platform. Section 2.2 introduces the multimodal fusion method based on hypergraph convolution, a key innovation for integrating diverse health data. Section 2.3 presents the algorithm framework, explaining how the hypergraph convolutional network is implemented. Section 3 presents the results and discussion, with Section 3.1 focusing on experiments conducted on benchmark datasets to validate the model’s performance, and Section 3.2 discussing experiments on a self-collected dataset to demonstrate real-world applicability. Finally, Section 4 summarizes the main findings, limitations, and future research work of this study.

## 2 Methodology

### 2.1 Platform architecture

**Figure 1:**
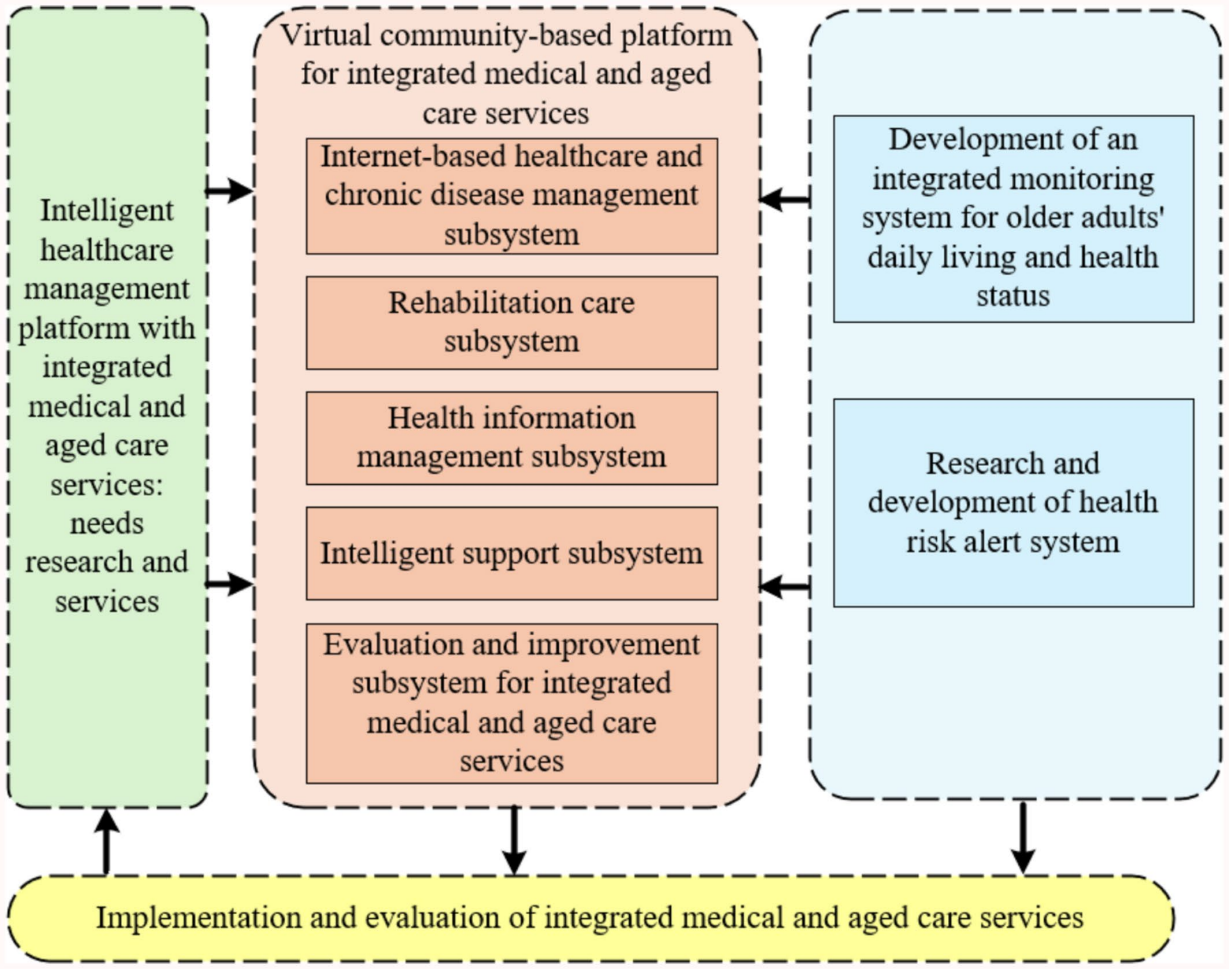
Architecture diagram of intelligent healthcare management platform with integrated medical and aged care services.

This study aims to develop an advanced and comprehensive intelligent healthcare management platform with integrated medical and aged care services for older adults. The platform fully utilizes the current popular Internet & big data technology to realize the efficient collection of diversified active health indexes of the older adults (20). It organically integrates medical services, health management, and older adults care services to build an integrated Internet intelligent service management system. Meanwhile, this study developed an electronic monitoring system based on medical IoT and mobile computing technologies for real-time monitoring of the living conditions and health status of the older adults, aiming to effectively intervene in their material and spiritual lives. With the core functions of recommending personalized health intervention programs and providing accurate risk warnings, the platform constructs an intelligent healthy aging mechanism that can be dynamically adjusted according to the health status of the older adults, and the mechanism is rigorously applied and verified. The architecture of the intelligent healthcare management platform with integrated medical and aged care services proposed in this study is shown in Figure 1.

#### 2.1.1 Demand research and design of services for intelligent healthcare management platform with integrated medical and aged care services

Based on the demand analysis of multiple subjects of healthcare and nursing services, this study promotes the construction of the intelligent service platform in accordance with the following paths: (1) By analyzing the functional demands of the core subjects of the intelligent platform, such as the older adults, medical institutions, senior care institutions, and the community, and by applying system design theory to translate stakeholder needs into modular functional components and functional modules. (2) Combining family-community-institution multi-scenario interaction characteristics, systematically sort out the common needs and personalized service boundaries of the six major fields such as healthy aging, medical rehabilitation, and psychological support. (3) Furthermore, the rising prevalence of chronic diseases among the older adults, such as cancer, underscores the need for integrated medical and aged care services. As highlighted by Song et al. (21), the management of cancer survivors in China requires a holistic approach that combines medical treatment with supportive care services. Our proposed platform aims to address this need by providing a comprehensive solution for older adults healthcare management.

#### 2.1.2 Development of a virtual community-based integrated medical and aged care services platform

This study constructs a community virtual healthcare integration cloud platform based on the three-layer architecture of “monitoring terminal - cloud platform - management terminal.” The specific implementation path is as follows: (1) Establish a standardized medical and health data interface, integrate the core subsystems of chronic disease management, rehabilitation care, health information management, etc., and form a mobile medical service system with doctor-patient collaboration. (2) Complete the development of the life and health status monitoring and warning system by defining the health risk warning task set for the older adults and conducting modeling and analysis, and realizing the functions of collecting, transmitting and storing monitoring data. (3) Develop the functional modules of the health risk alarm system, focus on breaking through the key technical links such as unified push and feedback processing of alarm information, and put them into practical application after system testing and verification. The platform realizes closed-loop management of service evaluation and dynamic optimization through a multi-system linkage mechanism.

#### 2.1.3 Application and evaluation of community and home-based integrated medical and aged care services

This study promotes the R&D and application of the platform through a three-phase validation system: (1) Based on the multi-dimensional satisfaction surveys of the older adults, co-living persons, health care team and service providers, combined with expert demonstration and systematic evaluation, comprehensively evaluates the clinical feasibility, operational effectiveness, data security and health economic benefits of the health care intelligent service platform. (2) Focus on the ECG monitoring module, and verify the timeliness of monitoring response, accuracy of diagnostic results, and intervention effectiveness of cardiovascular and cerebrovascular disease risk early warning through continuous data collection and retrospective analysis of clinical cases; (3) Classify the older adults population into three categories: supportive, protective, and healthy according to the economic stratification model, and explore the adaptive paths and sustainable mechanisms of differentiated operation modes by simulating the cost–benefit of the services. Through service cost–benefit simulation, explore the adaptation path and sustainable mechanism of differentiated operation mode.

#### 2.1.4 Technical route

**Figure 2:**
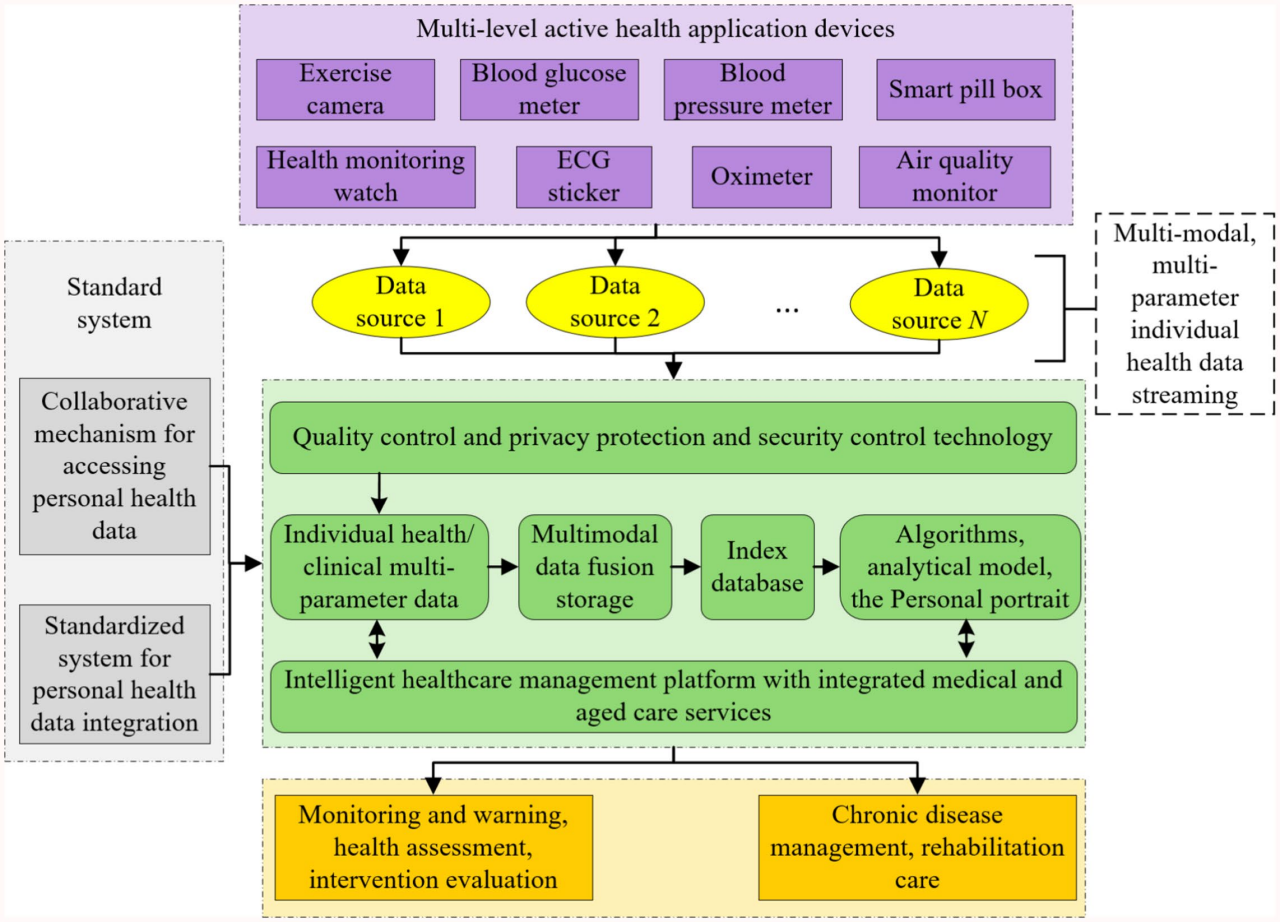
Technology roadmap of intelligent healthcare management platform with integrated medical and aged care services.

This study follows the technical route of “standard specification-data processing-algorithm modeling-platform validation,” and promotes the construction of intelligent healthcare management platform with integrated medical and aged care services in stages. The detailed roadmap is shown in Figure 2.

This study promotes the construction of intelligent healthcare management platform with integrated medical and aged care services with the following technology paths:

(1) Privacy and security system construction: establish a hierarchical protection mechanism for personal health monitoring data, and based on the asymmetric encryption technology and the private key management system, realize dynamic control of health record access privileges and security auditing;
(2) Multimodal data collection technology: develop integrated wearable devices and supporting software systems to support real-time collection and transmission of physiological parameters such as blood pressure, blood glucose, ECG, and multi-dimensional health information such as behavior and psychology, and to construct active health monitoring data streams;
(3) Heterogeneous data governance scheme: design a standardized model of health data under the framework of spatio-temporal association, adopt Solr/ElasticSearch technology to realize the metadata definition and efficient indexing of multi-source heterogeneous data, and rely on HBase/Hive/Blockchain technology to establish a unified storage and association mechanism of unstructured data.
(4) Intelligent algorithm model development: develop a new generation of artificial intelligence algorithms and models with the functions of disease early warning, lifestyle intervention, psychological and behavioral advice, and personalized nutritional recommendation. Construct an online learning framework that integrates convolutional neural networks, recurrent neural networks and evolutionary algorithms, develop relocatable models with functions such as disease early warning, behavioral intervention, nutritional recommendations, etc., and realize lightweight transplantation of algorithms from cloud training to mobile deployment.
(5) Platform system integration verification: build a multimodal computing service platform based on the Spark framework, construct a medical knowledge map through NLP technology, develop a clinical application system covering the core functions of assessment and analysis, prediction and early warning, and intervention and evaluation, and establish a “monitoring-diagnosis-intervention” closed loop management mechanism.

#### 2.1.5 Functional design

**Figure 3:**
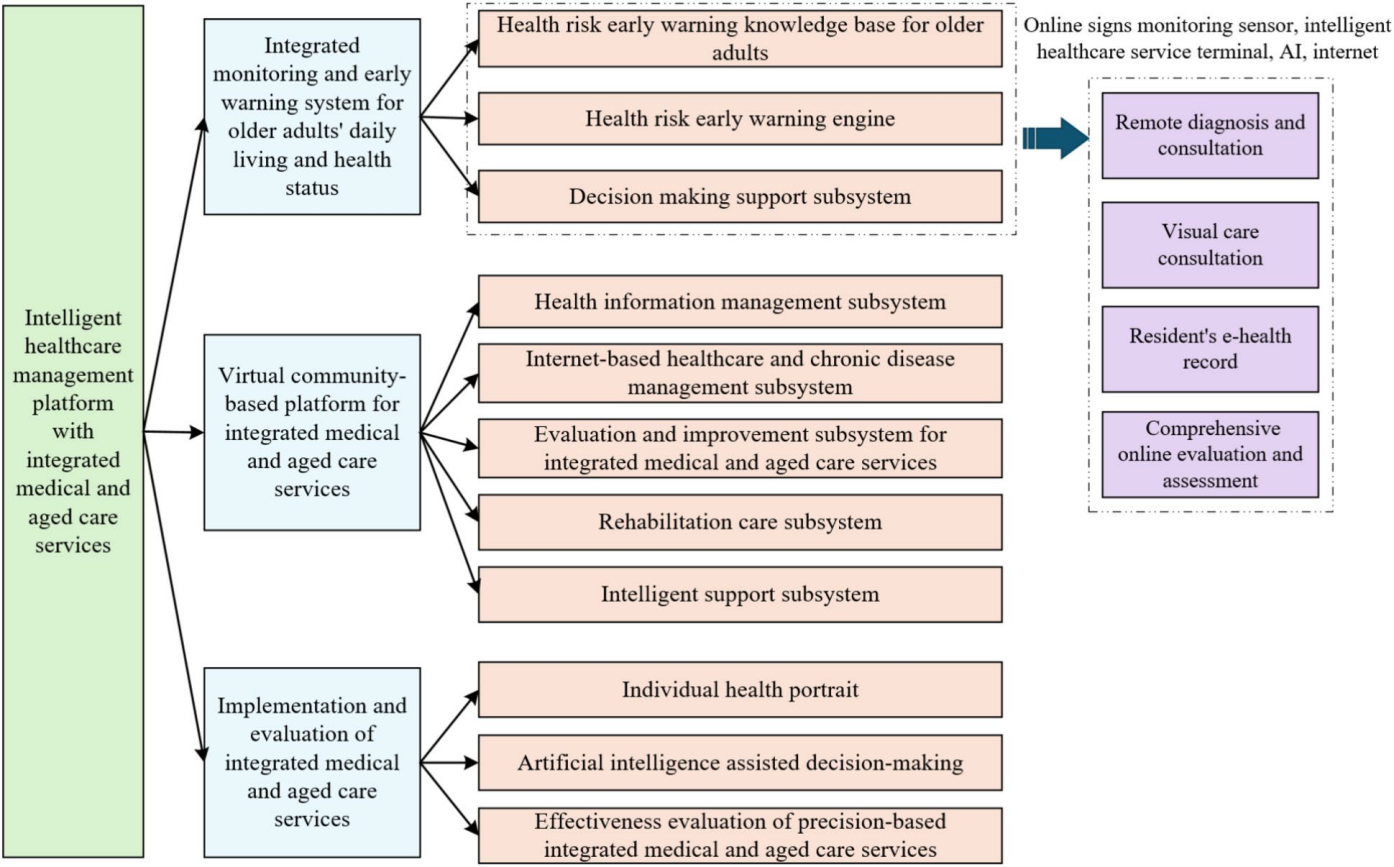
Functional framework of the intelligent healthcare management platform with integrated medical and aged care services.

Based on the Internet technology architecture, this study builds an intelligent service system around the active health management needs of the older adults, with the following specific technical realization paths: (1) develop a medical and nursing knowledge base system and a cloud platform that integrates the management of chronic diseases and rehabilitation services, and integrate deep learning algorithms to realize health data analysis and decision support; (2) develop a multimodal risk prediction system that focuses on the major health risks of cardio-cerebrovascular events and metabolic abnormalities. For major health risks such as cardiovascular and cerebrovascular events, metabolic abnormalities, etc., we develop multimodal risk prediction algorithms and dynamic intervention models; (3) through the construction of personalized health profiles and intelligent program generation technology, customized health management paths are formed to effectively alleviate the pain point of the industry, which is the shortage of professional manpower. The functional framework of the intelligent healthcare management platform with integrated medical and aged care services is shown in Figure 3.

The Hypergraph Convolutional Network (HGCN) model, integral to the proposed framework, entails substantial computational resource requirements due to the complexity of hypergraph convolution operations and multimodal data processing. Experimental evaluations were performed on a server configured with an NVIDIA RTX 3090 GPU, 64 GB of RAM, and an Intel i9-10900K CPU. The computational demand scales linearly with the number of nodes (representing patients) and hyperedges (capturing multimodal relationships) in the hypergraph. To address scalability, the framework employs parallelization techniques and optimization strategies, including distributed computing and model pruning, to maintain performance efficiency as the population size increases.

Integrating our platform into existing healthcare infrastructures presents several challenges. First, ensuring interoperability with diverse electronic health record (EHR) systems requires adherence to standardized data formats, such as HL7 FHIR, to facilitate seamless data exchange. Second, compliance with data privacy regulations (e.g., HIPAA, GDPR) is critical; our platform incorporates robust encryption, access controls, and anonymization techniques to protect sensitive health data, as outlined in Section 2.1.4. Third, user adoption hinges on intuitive interfaces and minimal training requirements, which we address through user-centered design and comprehensive documentation. Finally, integrating with legacy systems may require custom APIs or middleware, necessitating close collaboration with healthcare IT teams to ensure smooth deployment. These considerations ensure that our platform is practically deployable and scalable for real-world older adults healthcare management.

### 2.2 Multimodal fusion method based on hypergraph convolution and combining with underlying feature learning

In order to mine complex disease associations more efficiently and to improve the accuracy of personalized services for the older adults, a HGCN is introduced in this study. HGCN, with its powerful explicit modeling capability of complex relationships, can extend the medical knowledge graph into a dynamic spatio-temporal graphs. It is capable of mapping heterogeneous data such as physiological indicators, electronic medical records, and environmental parameters into a unified graph representation. This mapping approach enables it to provide better solutions in several key scenarios such as disease path discovery, multimodal data alignment, and privacy-preserving collaborative computing.

#### 2.2.1 Hypergraph concepts

A hypergraph is a structure in graph theory distinguished by its edges, known as hyperedges, which can connect any number of vertices, unlike traditional graphs where edges are limited to connecting exactly two vertices. Compared to traditional graphs, hypergraphs offer greater flexibility in modeling higher-order relationships, such as group structures in social networks or multi-molecule interactions in bioinformatics.

**Figure 4:**
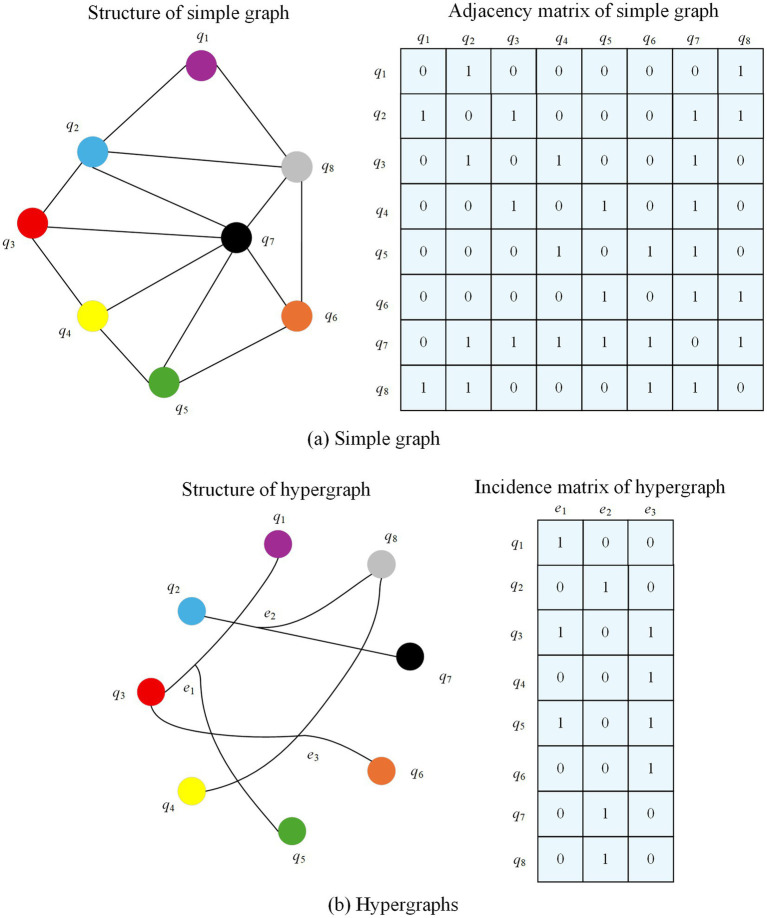
Comparison between simple graph and hypergraph representations for movie-actor relationships.

This study illustrates the evolutionary logic of the graph model through the association relationship between movies and actors. In the traditional graph structure, if a node is set to be a movie and an edge is set to be an actor, the connection of edges indicates that the corresponding actor has acted in two movies, and the absence of edges indicates that no actor has acted in two movies. However, an edge in a traditional graph can only connect two nodes, which makes it difficult to visually present all the movies in which an actor has acted, resulting in redundant edge information. As an extension of traditional graph, hypergraph has the advantage that one edge can connect multiple nodes, which can visualize all the works of actors. While simple graph focuses on the neighbor relationship between nodes, hypergraph is better at characterizing the complex association between nodes, and has an incomparable advantage in expressing relevance in traditional graphs. Figure 4 shows the comparison between simple graph and hypergraph for movie-actor relationship. Simple graph can be represented in the form of adjacency matrix, if there are edges between nodes, the matrix element corresponds to 1, and vice versa is 0. From the comparison figure, it can be seen that the simple graph connects movies based on shared actors, requiring many pairwise edges (14 in this case), which results in redundancy and poor scalability. In contrast, the hypergraph directly connects each actor to all movies they acted in via a single hyperedge, reducing edge complexity (3 edges total) and capturing higher-order relationships. This illustrates the advantage of hypergraphs in modeling group associations, which is especially important in multimodal healthcare data.

Given a hypergraph A=(Q,E,M), $Q=\{q_{1},q_{2},\cdots ,q_{|q|}\}$ denotes the set of nodes, $E=\{e_{1},e_{2},\cdots ,e_{|E|}\}$ denotes the set of hyperedges, and *M* is the diagonal matrix. Its diagonal element $m(e_{y})$ denotes the weight of a hyperedge $e_{y}\in E$. The hypergraph can be represented by the association matrix *B* of ∣Q∣×∣E∣. A matrix element $b(q_{x},e_{y})=1$ if node $q_{x}\in Q$ exists in a hyperedge $e_{y}\in E$ and vice versa. The degree of node $q_{x}\in Q$ is defined as $d(q_{x})=\sum_{e_{y}\in E}\;m(e_{y})b(q_{x},e_{y})$ and the degree of edge $e_{y}\in E$ is $d(e_{y})=\sum_{q_{x}\in Q}\;b(q_{x},e_{y})$. $D_{q}$ and $D_{e}$ are the diagonal matrices of the node degree and the degree of the hyperedge, respectively. The Laplacian matrix of the hypergraph can be expressed as $L^{A}=X-D_{Q}^{-1/2}\mathrm{BM}D_{e}^{-1}B^{N}D_{Q}^{-1/2}$, and $L^{A}$ is semi-positive definite.

#### 2.2.2 Hypergraph convolution

Similar to graph convolution, hypergraph convolution is also categorized into two ways: spectral domain convolution and null domain convolution. For hypergraph spectral domain convolution, the convolution operation is performed in the spectral domain of the graph. For hypergraph null domain convolution, the convolution operation aggregates neighbor information on the hypergraph. The information is propagated from the node to the hyperedge and from the hyperedge back to the node, thus completing the information transfer.

##### 2.2.2.1 Hypergraph spectral domain convolution

Given a hypergraph A=(Q,E,M) with the number of nodes ∣Q∣. The hypergraph Laplacian matrix $L^{A}$ is a ∣Q∣×∣Q∣ semi-positive definite matrix. The orthogonal eigenvector matrix *P* and the diagonal matrix $\Lambda =\mathrm{diag}(\lambda_{1},\cdots ,\lambda_{|Q|})$ containing non-negative eigenvalues can be obtained from the Eigen decomposition $L^{A}=P\Lambda P^{N}$. For a signal $i\in {\mathbb{R}}^{|Q|}$ on the hypergraph, *i* can be considered as a vector containing the features of all nodes. Where each node is represented by a scalar. Define its Fourier variation as $\hat{i}=P^{N}i$, the corresponding eigenvector is the Fourier basis and the eigenvalues are the frequencies. The spectral domain convolution of the signal *i* with the filter g can be expressed as:


(1) $$a⋆i=P[(P^{N}a)⊙(P^{N}i)]=\mathrm{Pa}(\Lambda )P^{N}i$$

Where ⋆ denotes the convolution operator, ⊙ denotes the Hadamard product of elements, and a(Λ) is a function of the eigenvalues of $L^{A}$.

However, the above equation is too computationally intensive and has a computational complexity of O(∣Q∣2). To solve this problem, a(Λ) can be approximated by a K-order polynomial, which can be obtained by using K-order truncated Chebyshev polynomials here:


(2) $$a(\Lambda )\approx \sum_{z=0}^{Z}\;\theta_{z}N_{z}(\tilde{\Lambda })$$

Where $\tilde{\Lambda }=\frac{2}{\lambda_{\max}}\Lambda -X$, $\lambda_{\max}$ denote the largest eigenvalue in $L^{A}$. $\theta_{z}$ is the Chebyshev coefficient. The recursive formula for the Chebyshev polynomials is $N_{z}(i)=2iN_{z-1}(i)-N_{z-2}(i)$, where $N_{0}(i)=1$ and $N_{1}(i)=i$. Substituting Equation 2 into Equation 1 yields:


(3) $$a⋆i\approx P\sum_{z=0}^{Z}\;\theta_{z}N_{z}(\tilde{\Lambda })P^{N}i=\sum_{z=0}^{Z}\;\theta_{z}N_{z}(P\tilde{G}P^{N})i=\sum_{z=0}^{Z}\;\theta_{z}N_{z}({\tilde{L}}^{A})i$$

Where ${\tilde{L}}^{A}=\frac{2}{\lambda_{\max}}L-X$. After approximation, as shown in Equation 3, it can be found that the computation of Laplacian eigenvectors is replaced with only matrix operations, improving the computational complexity. Because the Laplacian of the hypergraph itself can already represent the higher-order relationships between nodes well, *Z* = l limits the order of the convolution. Meanwhile, due to the scale adaptation of the neural network, it can be approximated so that $\lambda_{\max}\approx 2$, then the convolution operation can be further simplified as:


(4) $$a⋆i\approx \theta_{0}i-\theta_{1}D_{q}^{-1/2}\mathrm{BM}D_{e}^{-1}B^{N}D_{q}^{-1/2}i$$

Where $\theta_{0}$ and $\theta_{1}$ are the filter parameters for all nodes. In order to avoid the overfitting problem, let $\theta_{0}=\frac{1}{2}\theta D_{Q}^{-1/2}BD_{e}^{-1}B^{T}D_{Q}^{-1/2}$, $\theta_{1}=-\frac{1}{2}\theta$ and Equation 4 reduces to Equation 5:


(5) $$a⋆i\approx \frac{1}{2}\theta D_{q}^{-1/2}B(M+X)D_{e}^{-1}B^{N}D_{q}^{-1/2}i\approx \theta D_{q}^{-1/2}\mathrm{BM}D_{e}^{-1}B^{N}D_{q}^{-1/2}i$$

Where M+X can be considered as the hyperedge weights, and here *M* is initialized as a unit array. When the hypergraph node feature matrix $I_{Q}^{l}$ of layer *l* is given, the hypergraph convolution can be expressed as shown in Equation 6:


(6) $$I_{Q}^{l+1}=\sigma (D_{q}^{-1/2}\mathrm{BM}D_{e}^{-1}B^{N}D_{q}^{-1/2}I_{Q}^{l}\Theta^{l})$$

Where $\Theta^{l}\in {\mathbb{R}}^{d_{1}\times d_{n+1}}$ is the learnable parameter of the *l*-th layer.

##### 2.2.2.2 Hypergraph null domain convolution

For a hypergraph A=(Q,E,M), assume that $I_{Q}$ is its node feature matrix, $I_{Q}\in {\mathbb{R}}^{|Q|\times d}$, *d* is the feature dimension, and $i_{q_{x}}\in I_{\mathrm{QV}}$ denotes the feature vector of node $q_{x}$. Define the set of hyperedges containing node $q_{x}$ as $E(q_{x})=\{e_{y}\in E|q_{x}\in e_{y}\}$. The information transfer from node $q_{x}$ to the hyperedges at layer *l* can be represented as


(7) $$i_{e_{y}}^{l}=\text{Messag}e_{1}(\{i_{q_{v}}^{l}|q_{v}\in e_{y},q_{x}\in e_{y}\})$$

As shown in Equation 7, where $q_{v}$ is all nodes within the specified hyperedge ej containing $q_{x}$. $i_{e_{y}}^{l}$ is the feature of hyperedge $e_{y}$ of layer *l*, $i_{e_{y}}^{l}\in {\mathbb{R}}^{d_{x}}$, obtained from the node features by the aggregation function $\;{\text{Message}}_{1}(\cdot )$.$d_{l}$ refers to the dimension of the hyperedge features of layer *l*. The transfer of information from a hyperedge to a node $q_{x}$ can be expressed as shown in Equation 8:


(8) $$i_{q_{x}}^{l+1}=\text{Update}(i_{q_{x}}^{l},\text{Messag}e_{2}(\{m(e_{y})\cdot i_{e_{y}}^{l}|e_{y}\in E(q_{x})\}))$$

Update(•)denotes the update function of the node features from layer *l* to piece *l* + 1. The expressions for the aggregation function and update function are as follows:


(9) $$\text{Messag}e_{1}(\{i_{q_{v}}^{l}|q_{v}\in e_{y},q_{x}\in e_{y}\})=\sum_{q_{v}\in e_{y},q_{x}\in e_{y}}\;\frac{i_{q_{v}}^{l}}{d(e_{y})}$$

(10) $$\text{Messag}e_{2}(\{m(e_{y})\cdot i_{e_{y}}^{l}|e_{y}\in E(q_{x})\})=\sum_{e_{y}\in E(q_{x})}\;m(e_{y})\cdot i_{e_{y}}^{l}$$

(11) $$\begin{array}{l} \text{Update}(i_{q_{x}}^{l},\text{Messag}e_{2}(\{i_{e_{y}}^{l}|e_{y}\in E(q_{x})\}))=\\ \frac{\text{Messag}e_{2}(\{i_{e_{y}}^{l}|e_{y}\in E(q_{x})\})}{|E(q_{x})|}\cdot \Theta^{l} \end{array}$$

According to Equations (9–11) the information transfer can be divided into 3 steps. First, select a hyperedge $e_{y}$ that contains node $q_{x}$. Sum the features of all nodes in hyperedge $e_{y}$ and take the average as its edge features. The other hyperedges containing node $q_{x}$ are processed in the same way to obtain the features of all hyperedges in the hyperedge set $E(q_{x})$. Next, the hyperedges in the hyperedge set $E(q_{x})$ have different weights, and the features of the hyperedges are multiplied with their corresponding weights as the total features of all hyperedges within the hyperedge set $E(q_{x})$. Finally, the total features of the hyperedge set $E(q_{x})$ are averaged and multiplied with a transformation parameter as the features of node $q_{x}$ after information transfer at that layer.

Extending the node’s information transfer to the whole hypergraph, the null domain convolution form of the hypergraph can be obtained:


(12) $$I_{Q}^{l+1}=\sigma_{1}(D_{q}^{-1}\mathrm{BM}D_{e}^{-1}B^{N}I_{Q}^{l}\Theta^{l})$$

Where *B* is the hypergraph association matrix and $\sigma_{1}(\cdot )$ is the nonlinear activation function. Let $I_{E}^{l}$ denote the hyperedge feature matrix of layer *l*. Then $I_{E}^{l}=MD_{e}^{-1}B^{N}I_{Q}^{l}$ denotes feature propagation from node to hyperedge. $I_{Q}^{l+1}=D_{Q}^{-1}BI_{E}^{l}$ denotes feature propagation from hyperedge to node.

#### 2.2.3 Algorithm framework

**Figure 5:**
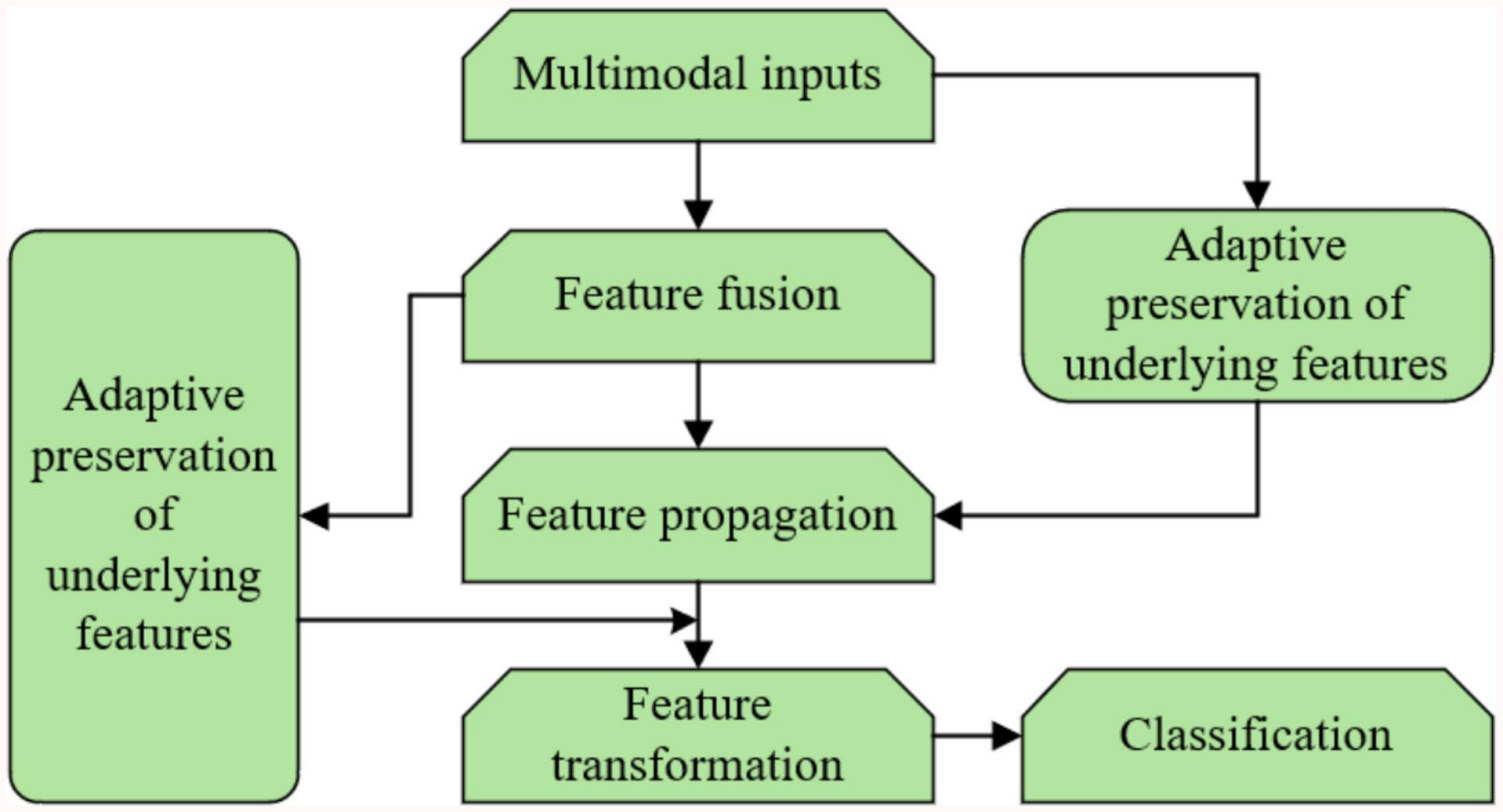
Block diagram of the proposed multimodal hypergraph convolutional model with adaptive feature learning.

Figure 5 presents the block diagram of the proposed multimodal hypergraph convolutional model, which integrates three key components: feature propagation, adaptive retention of underlying features, and feature transformation. The process begins with the construction of a multimodal hypergraph from the input data, where nodes represent entities (e.g., patients) and hyperedges capture higher-order relationships across different modalities (e.g., physiological, behavioral, environmental). The multimodal inputs are then fused at the feature level to form the underlying features of the hypergraph nodes. These features are passed through two parallel paths: (1) Feature propagation, where information is aggregated across hyperedges to capture high-level representations of the nodes; (2) Adaptive retention of underlying features, where the model learns node-specific weights to preserve important low-level information that might be diluted during propagation. The high-level and adaptively retained low-level features are then combined and passed through a feature transformation layer (a fully connected network) for dimensionality reduction and classification. This design ensures that both the complex relationships captured by the hypergraph and the individual characteristics of the nodes are effectively utilized in the final prediction.

The proposed rooted hierarchical feature learning algorithm is central to the adaptive retention of underlying features and operates hierarchically to balance high-level and low-level feature integration. Its architecture comprises two main modules: (1) a feature propagation module, implemented via hypergraph convolution, which aggregates multimodal information to form high-level representations, and (2) an adaptive retention module, which employs a learnable diagonal matrix to assign node-specific weights to low-level features (e.g., raw physiological signals like ECG peaks). The training routine optimizes these weights using a multi-task loss function combining cross-entropy for classification and a regularization term to preserve feature fidelity, trained over 600 epochs with the Adam optimizer (learning rate 0.001, weight decay 0.0005). Key hyperparameters include two feature propagation layers, a dropout rate of 0.5 to prevent overfitting, and a hidden layer dimension of 128, all tuned via cross-validation on the UCI dataset. Data mapping involves concatenating multimodal features (physiological, behavioral, environmental) into a unified node feature matrix before processing, ensuring seamless integration across modalities. This hierarchical design enhances classification accuracy by retaining critical low-level details while leveraging high-level patterns.

#### 2.2.4 Multimodal hypergraph

In a multimodal scenario, nodes have different feature representations in different modalities, let $I_{Q(w)}$ denote the node feature matrix in modality *w*, $I_{Q(w)}\in {\mathbb{R}}^{|Q|\times d(w)}$, w∈{1,2,⋯,W}, and *W* is the number of modalities. The multimodal representation of the node identity matrix is


(13) $$I_{Q}=\text{Concat}(\{I_{Q(w)}|w\in \{1,2,\cdots ,W\}\})$$

Where Concat(•)denotes the corresponding row cascade. In the feature space represented by modality *w*, the hypergraph can be constructed according to KNN to obtain the association matrix B(w), and then the multimodal hypergraph can be represented as


(14) B=Concat({B(w)∣w∈{1,2,⋯,W}})

Equations 13 and 14 are fed into the feature propagation as the outputs of the multimodal inputs and the multimodal hypergraph construction.

Nodes represent individual patients (e.g., the 50 participants in our self-collected dataset), with each node linked to multimodal features (physiological, behavioral, environmental) at specific time points. Hyperedges connect groups of patients sharing similar health patterns across these modalities, forming higher-order relationships that evolve temporally. For example, a hyperedge might connect patients with similar heart rate trends and activity levels over a week. The spatio-temporal aspect arises as hyperedges are dynamically updated with time-series data, capturing health state changes (e.g., disease progression). Weights on hyperedges are adaptively assigned using two criteria: (1) temporal proximity, and (2) feature similarity. This adaptive weighting ensures the hypergraph reflects both spatial (cross-patient) and temporal (within-patient) dynamics, enhancing its ability to model complex health patterns for older adults care.

#### 2.2.5 Multimodal hypergraph convolution

Equations 6, 12 show a complete layer of hypergraph convolution. In order to separate the feature propagation from the feature transformation and also to remove the nonlinear part, it is modified as


(15) $$I_{Q}^{l+1}=D_{Q}^{-1/2}\mathrm{BM}D_{e}^{-1}B^{N}D_{q}^{-1/2}I_{Q}^{l}$$

(16) $$I_{Q}^{l+1}=D_{q}^{-1}\mathrm{BM}D_{e}^{-1}B^{N}I_{Q}^{l}$$

It can be found from Equations 15, 16, both the spectral domain convolution and the null domain convolution conform to the form $I_{Q}^{l+1}=GI_{Q}^{l}$. For spectral domain convolution, $G=D_{q}^{-1/2}\mathrm{BM}D_{e}^{-1}B^{N}D_{q}^{-1/2}$and for null domain convolution, $G=D_{q}^{-1}\mathrm{BM}D_{e}^{-1}B^{N}$. Defining *G* as a feature aggregator, the feature propagation can be expressed as $I_{Q}^{l+1}=GI_{Q}^{l}$. Unlike simple graph that add node self-loops, hypergraphs constructed according to KNN come with self-connections of nodes. However, since the hyper edges represent higher-order relations and contain more node information than simple edges, the role of the underlying information is easily diluted after convolutionally learning the high-level representation of the nodes.

Equation 11 ignores the features of node $q_{x}$ at layer *l* when updating its layer *l* + 1 features. When the number of layers increases, the node’s underlying features $i_{q_{x}}^{0}$ are masked and the model tends to be oversmoothed. To solve this problem, some of the underlying features are retained after feature propagation, and the final node feature matrix is obtained as shown in Equation 17


(17) $$I_{Q}^{\text{FNxt}}=G^{L}I_{Q}^{0}+\sigma_{2}(R)I_{Q}^{0}$$

Where *L* denotes the number of layers of feature propagation; $I_{Q}^{0}$ denotes the initial underlying feature matrix of the node; $I_{Q}^{\text{FNxt}}$ is the input of the feature transformation part, *R* is the learnable adaptive diagonal matrix, and $\sigma_{2}(\cdot )$ is the nonlinear activation function.

The multimodal node representation $I_{Q}^{0}$ is the node’s bottom layer feature, and the node’s high-level feature representation $I_{Q}^{L}$ is obtained by completing the information aggregation through *L* feature propagation layers. Meanwhile, in the adaptive retention of the bottom layer feature part, the network is trained to obtain the adaptive diagonal matrix *R*, and the diagonal elements are the corresponding node’s bottom layer feature weights. *R* is constrained by a nonlinear activation function, and then the node bottom layer features are multiplied with the corresponding weights as the node’s bottom layer feature representation, and the sum of the high-level features and bottom layer features is input to the feature transformation part. The feature transformation part is not labeled in the figure, and a fully connected layer is used so that the final output dimension is consistent with the number of node categories.

### 2.3 Data preprocessing

To ensure the quality and consistency of the data used in our experiments, we applied several preprocessing steps to both the UCI and PhysioNet datasets.

For the UCI datasets, which include the Heart Disease and Diabetes 130-US Hospitals subsets, we handled missing values using imputation techniques. In the Heart Disease dataset, missing values in categorical attributes such as ‘thal’ (thalassemia) and ‘ca’ (number of major vessels) were imputed using the mode of the respective columns. For the Diabetes 130-US Hospitals dataset, missing numerical values (e.g., laboratory results like HbA1c) were imputed using the mean of the available data, while missing categorical values (e.g., diagnosis codes) were assigned a default “Unknown” category.

For the PhysioNet datasets, which consist of time-series physiological signals, the preprocessing involved normalization, handling of missing values, and segmentation: (1) Normalization: All physiological signals (e.g., ECG, EEG) were normalized to have zero mean and unit variance. (2) Handling Missing Values: Missing segments in the time-series data were addressed using linear interpolation for gaps shorter than 5 s; longer gaps were excluded from the analysis. (3) Segmentation: The time-series data were segmented into fixed-length windows (e.g., 30-s epochs for the Sleep Heart Health Study) to facilitate feature extraction using Time-Series Convolutional Neural Networks (TS-CNN).

## 3 Result analysis and discussion

### 3.1 Experiments on benchmark datasets

#### 3.1.1 Dataset introduction

The experiments were conducted on two benchmark medical datasets: the UCI Machine Learning Repository’s health-related dataset and the PhysioNet multimodal physiological and clinical datasets. Detailed statistics are summarized in Table 1.

**Table 1:** UCI and PhysioNet dataset statistics.

Dataset	Total samples	Classes	Training samples	Testing samples	Mode
UCI-Heart Disease	303	2	242	61	TS-CNN/CFE
UCI-Diabetes 130-US	100,000	3	80,000	20,000	TS-CNN/CFE
PhysioNet-SHHS	5,804	5	4,643	1,161	TS-CNN/CFE
PhysioNet-MIT-BIH	48	5	38	10	TS-CNN/CFE

The UCI Dataset integrates two health-related subsets: the Heart Disease Dataset and the Diabetes 130-US Hospitals Dataset, collectively comprising 100,303 patient records. The Heart Disease subset includes 303 records with 14 attributes, such as physiological indicators (e.g., blood pressure, cholesterol levels, ECG features) and behavioral factors (e.g., smoking status), aimed at predicting heart disease presence. The Diabetes 130-US Hospitals subset contains 100,000 hospitalization records from diabetic patients, with 50 + attributes including laboratory results (e.g., HbA1c), medication records, and diagnosis codes. The combined UCI Dataset is split into 80,242 training samples and 20,061 testing samples, supporting tasks like chronic disease prediction and health status classification across multiple classes (e.g., disease severity or presence/absence).

The PhysioNet Dataset includes two subsets: the Sleep Heart Health Study (SHHS) and the MIT-BIH Arrhythmia Database. The SHHS dataset contains polysomnography (PSG) records from 5,804 participants, primarily middle-aged and older adults, with multimodal physiological signals (e.g., EEG, ECG, blood oxygen saturation) and behavioral annotations (e.g., sleep stages, apnea events). It is split into 4,643 training samples and 1,161 testing samples. The MIT-BIH Arrhythmia Database includes 48 half-hour ECG recordings from 47 patients, with annotations for heart rhythm abnormalities, divided into 38 training samples and 10 testing samples.

To represent the datasets’ features, two feature extraction methods were employed: TS-CNN and Clinical Feature Embeddings (CFE). TS-CNN processes temporal physiological signals (e.g., ECG, PSG) to capture dynamic patterns, while CFE transforms structured clinical data (e.g., laboratory results, diagnosis codes) into low-dimensional embeddings for downstream tasks.

#### 3.1.2 Benchmark experimental setup

The experiment uses 2 layers of feature propagation and 2 fully connected layers as feature transformations. The activation function $\sigma_{2}(\cdot )$ is set to be a sigmoid function, the dimension of the hidden layer is 128, the dropout is 0.5, the epoch is 600, and the loss function is a cross-entropy function. The learning rate is set to 0.001, the Adam optimizer is used to optimize the loss function, the weight decay is set to 0.000 5, the decay coefficient is 0.7, and the decay speed is 200. In order to ensure the consistency between the hypergraph structure and the graph structure, the experiments use the hypergraph unfolding method to convert the constructed hypergraph structure into the simple graph structure. The process of hypergraph unfolding was carried out through the implementation of the clique expansion technique. In this approach, each hyperedge connecting multiple nodes is transformed into a clique, where every pair of nodes within the hyperedge is connected by an edge in the simple graph. This transformation preserves the higher-order relationships captured by the hyperedges while allowing us to apply standard graph convolutional networks (GCNs) for comparison. Importantly, the node features and labels remained unchanged during this process, ensuring a fair evaluation across different models.

Each experiment was conducted 10 times using 10-fold cross-validation, with results averaged to ensure robustness and mitigate overfitting. Additionally, we conducted external validation using a held-out subset (10% of samples) of the UCI dataset, achieving an accuracy of 89.12%, closely aligning with our main result of 89.58%. This external validation demonstrates the model’s generalizability across different data distributions, reinforcing its robustness for real-world healthcare applications.

#### 3.1.3 Benchmark results and discussion

To evaluate the effectiveness of the proposed method, experiments were conducted on the health status classification task using the UCI and PhysioNet datasets. The results were compared with classic graph-based and hypergraph-based methods, including the miRNA-disease association prediction method by Liang et al. (22) and the multi-modal hypergraph neural network by Liu et al. (23). We also evaluated our method against a transformer-based model by Ilias et al. (24) and a standard multimodal CNN by Arora et al. (25), achieving accuracies of 86.78, 85.92, 87.45 and 88.23% on UCI, respectively, compared to our 89.58%. On PhysioNet, these models scored 82.14, 81.05, 80.39 and 81.46%, vs. our 84.52%. The comparison of experimental accuracy results is presented in Table 2.

**Table 2:** Comparison of experimental accuracy results.

Method	UCI (%)	PhysioNet (%)
Literature (22)	87.45	80.39
Literature (23)	88.23	81.46
Literature (24)	86.78	82.14
Literature (25)	85.92	81.05
Proposed	89.58	84.52

From Table 2, it is clear that the proposed method achieves the highest classification accuracy on both datasets, outperforming all compared methods. Specifically, on the PhysioNet dataset, the proposed method outperforms literature (23) by 3.06%, literature (22) by 4.13%, literature (24) by 2.38% and literature (25) by 3.47%. On the UCI dataset, the proposed method surpasses literature (23) by 1.35%, literature (22) by 2.13%, literature (24) by 2.80% and literature (25) by 3.66%. These results demonstrate the superior performance of the proposed method in enhancing classification accuracy. Key Innovations in the proposed method while literature (23) effectively aggregates neighbor information to model high-level features, it often neglects the low-level features inherent to individual nodes. The proposed method overcomes this limitation by introducing an adaptive mechanism that assigns weights to these low-level features, enabling more effective feature extraction. This adaptability is a key factor in its superior performance, as it leverages both high-order relationships and node-specific information to boost classification accuracy. In addition, we performed paired t-tests, confirming that our method’s improvements over all baselines are statistically significant (*p* < 0.05) on both datasets. These results demonstrate the superior performance of the proposed method in enhancing classification accuracy.

**Figure 6:**
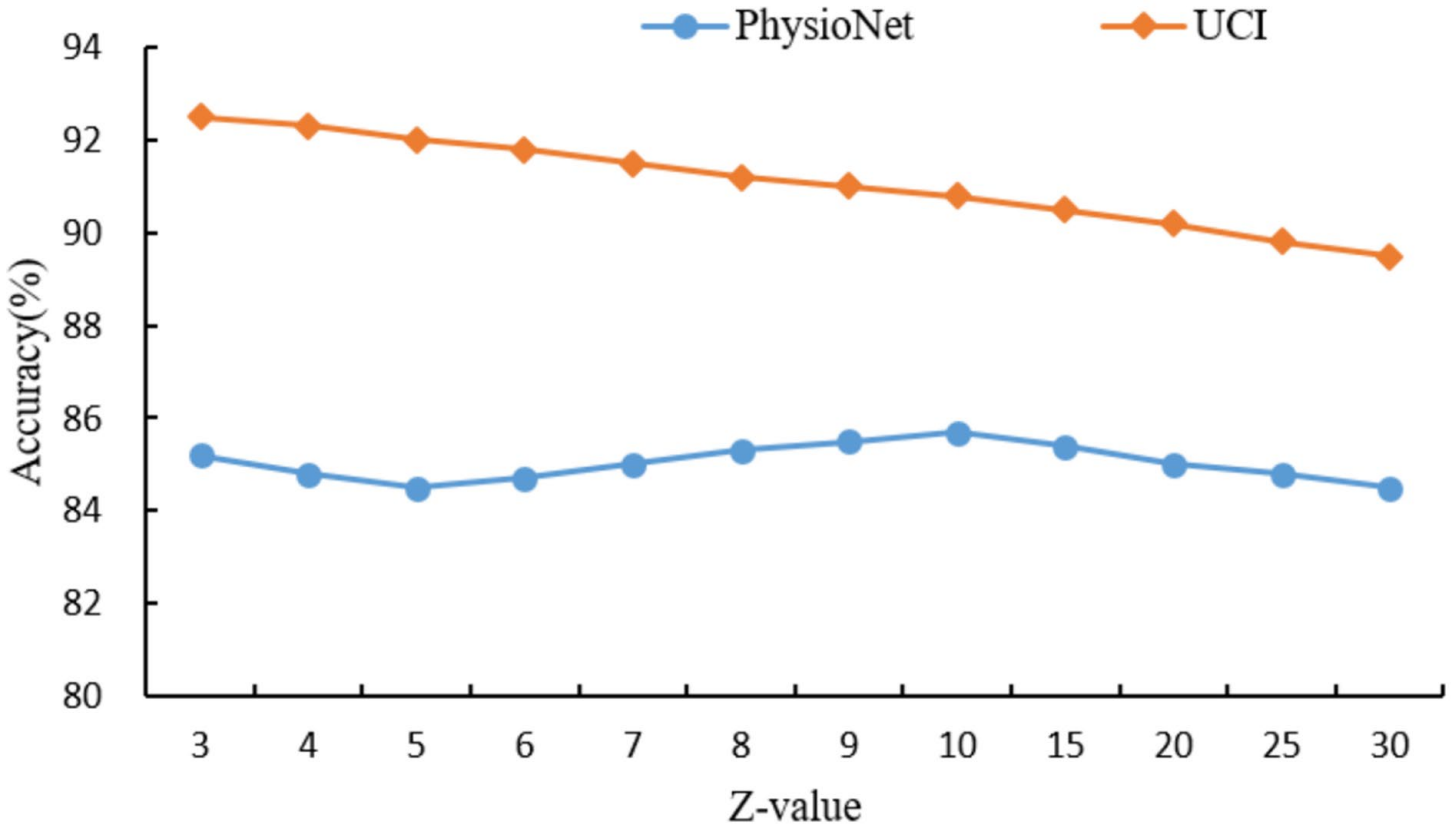
Effect of hyperedge size (Z) on classification accuracy across different datasets.

For the hypergraph construction, the choice of Z = 10 for the PhysioNet dataset and Z = 3 for the UCI dataset was determined through a grid search over a range of Z values ({3, 4, 5, 6, 7, 8, 9, 10, 15, 20, 25, 30}), as shown in Figure 6. The optimal Z values were selected based on the peak classification accuracy, balancing the capture of higher-order relationships with the risk of over-smoothing. For PhysioNet, Z = 10 was found to effectively capture complex multimodal interactions, as smaller Z values led to insufficient connectivity, while larger values diluted the specificity of hyperedges. For the UCI dataset, a smaller Z = 3 was optimal due to its larger size and denser data structure, where smaller hyperedges better preserved local relationships without introducing noise. Theoretically, this suggests that hyperedge size should scale with dataset complexity and node density to maintain meaningful higher-order connections. Similarly, for the self-collected dataset, K = 5 for KNN was chosen after evaluating K values from 3 to 10, where K = 5 provided a robust balance between capturing local neighborhood structures and avoiding excessive computational complexity, consistent with the dataset’s moderate size (50 participants). The dropout rate of 0.5 was selected to prevent overfitting, a common choice in deep learning models, as it effectively regularizes the network without overly compromising learned features, validated through cross-validation experiments. The choice of two feature propagation layers and two fully connected layers was guided by empirical results showing that this configuration maximized accuracy while maintaining computational efficiency, as additional layers led to diminishing returns and increased risk of over-smoothing. These selections were further validated by sensitivity analyses, ensuring robustness across datasets.

As can be seen in Figure 6, the hyperedge size Z, determined by the KNN parameter, controls the number of nodes connected by each hyperedge, thereby influencing the model’s ability to capture higher-order relationships. For the PhysioNet dataset, which contains multimodal physiological signals from a moderate number of participants, the classification accuracy peaks at Z = 10, achieving 85.7%. This suggests that Z = 10 optimally balances the capture of complex interactions without over-generalizing. For Z10, accuracy decreases as larger hyperedges dilute the specificity of relationships. In contrast, for the larger UCI dataset, which includes extensive patient records, the highest accuracy of 92.5% is achieved at Z = 3. This indicates that smaller hyperedges are more effective in preserving local relationships in dense datasets, while larger Z values introduce noise and reduce accuracy. These results highlight the importance of tuning Z based on dataset size and complexity to maximize the hypergraph’s ability to model meaningful higher-order relationships.

In order to verify the performance advantages of the intelligent healthcare management platform with integrated medical and aged care services, this paper conducts performance tests in terms of the maximum number of concurrent users and the system response time. Among them, the maximum number of concurrent users is an important index of system testing, which mainly characterizes the user carrying capacity and performance stability of the system. During the test, the system load is increased by gradually increasing the number of concurrent users until the system becomes overloaded. The performance indicators under different maximum concurrent users are shown in Table 3.

**Table 3:** Performance indicators for different maximum concurrent users.

Number of concurrent users	Average click rate (Hit/s)	Average execution rate (T/s)	Average execution time (s)	Request rate (%)
60	125.67	18.34	0.038	100
120	158.92	20.45	0.045	100
180	175.36	22.18	0.052	100
240	165.84	24.76	0.058	100
300	150.27	30.59	0.065	100
360	135.48	35.22	0.072	100

As shown in Table 3, the system achieves a 100% request success rate under varying numbers of concurrent users. This indicates that the system can operate efficiently and stably without becoming overloaded. Furthermore, when the number of concurrent users reaches 360, the system achieves a peak average transaction rate of 35.22 transactions per second (T/s), with an average execution time of only 0.072 s and minimal variation. In summary, the platform demonstrates excellent performance in testing, maintaining a 100% request success rate and a low average execution time even with a high number of concurrent users. This confirms the platform’s superior performance and stability, ensuring it can meet the needs of large-scale concurrent users.

In order to evaluate the response performance of the platform proposed in this paper, we conducted a comprehensive test on it. The test is conducted by logging into the system simultaneously with multiple user accounts on different devices, and recording the changes of various performance indicators, the results are shown in Table 4.

**Table 4:** Test results of system transaction response time, server port traffic and packet loss rate.

Number of concurrent users	Minimum response time (s)	Average response time (s)	Server port traffic (MB/s)	Packet loss rate (%)
60	3.0	4.0	50	0.001 ± 0.0005
120	3.1	4.2	70	0.001 ± 0.0005
180	3.2	4.3	90	0.001 ± 0.0005
240	3.3	4.5	110	0.001 ± 0.0005
300	3.4	4.6	130	0.001 ± 0.0005
360	3.5	4.8	150	0.001 ± 0.0005
420	3.6	5.0	170	0.002 ± 0.0007
480	3.7	5.2	190	0.003 ± 0.0008

The packet loss rate is reported with 95% confidence intervals derived from repeated stress tests.

Table 4 illustrates that as the number of concurrent users increases from 60 to 480, the system’s transaction response time exhibits only a modest increase, highlighting its robust performance under varying loads. With 60 concurrent users, the minimum response time is 3.0 s, and the average response time is 4.0 s. By the time the user count reaches 360, these metrics rise to 3.5 s and 4.8 s, respectively—an increase of just 0.5 s and 0.8 s. This increment is significantly below industry norms, demonstrating the system’s efficient response capability under high load.

Further experiments were conducted with 420 and 480 concurrent users to evaluate the system’s scalability beyond the initial 360-user threshold. At 420 users, the minimum response time increases to 3.6 s, and the average response time reaches 5.0 s. At 480 users, these values further rise to 3.7 s and 5.2 s, respectively. These results indicate that even at higher user loads, the system maintains low response times, with increments remaining manageable and well-suited for large-scale healthcare applications.

Server port traffic also scales steadily with the number of users, growing from 50 MB/s at 60 users to 150 MB/s at 360 users—an increase of 100 MB/s. With the additional tests, traffic rises to 170 MB/s at 420 users and 190 MB/s at 480 users. This consistent, linear progression underscores the system’s strong data throughput capacity, enabling it to handle large-scale concurrent access without performance bottlenecks.

The packet loss rate remains exceptionally low across all tested scenarios, further affirming the system’s reliability. From 60 to 360 users, the packet loss rate holds steady at 0.001% (±0.0005%). At 420 users, it increases slightly to 0.002% (±0.0007%), and at 480 users, it reaches 0.003% (±0.0008%). These values, supported by 95% confidence intervals from repeated stress tests, demonstrate that the system sustains high data integrity even under extreme conditions. The minimal rise in packet loss at higher user counts remains well within acceptable limits for healthcare systems, where reliability is paramount.

### 3.2 Self-collected dataset experiments

In order to validate the applicability of the proposed HGCN framework in real healthcare scenarios, especially for older adults health management, a self-collected dataset is constructed and used in this study. This section describes in detail the construction process, experimental design, and result analysis of this dataset, demonstrating the potential of HGCN in handling multimodal health data of the older adults.

#### 3.2.1 Dataset introduction

The self-collected dataset was collected through a two-month short-term pilot study in collaboration with a local elder care facility. The data came from 50 older participants aged between 65 and 85 years old and covered three main modalities: physiological indicators, behavioral logs, and environmental parameters that are essential for health monitoring in older adults. In terms of ethical approval, the study was conducted in accordance with the ethical standards of the institutional review board (IRB), and written informed consent was obtained from all participants prior to data collection. Data anonymization was ensured by removing all personally identifiable information and assigning unique codes to each participant’s data. The dataset was stored securely and accessed only by authorized personnel involved in the study.

Physiological data included continuous measurements of heart rate, blood pressure, and oxygen saturation via wearable devices (e.g., smartwatches and pulse oximeters), which are time-series data that reflect the participants’ cardiovascular health. Behavioral data included daily activity levels (e.g., steps, exercise intensity) and sleep patterns (e.g., duration, quality), also recorded through wearable devices, as well as participant self-reported dietary habits and medication adherence logs. Environmental data, on the other hand, is collected through IoT sensors placed in the participant’s living environment, including ambient temperature and humidity, factors that may influence health outcomes in older adults.

The dataset was designed to support disease risk prediction tasks, with a specific focus on early detection of cardiovascular events, a common health problem among older adults. Each participant was labeled as being in one of three risk categories: low, moderate, or high, based on clinical assessment. The dataset was divided into 40 training samples and 10 test samples to ensure a balanced distribution of risk categories. Despite the small size of this dataset, it provides a proof of concept for the ability of the HGCN framework to process real-world multimodal health data, complementing the experiments based on the benchmark dataset and providing domain-specific validation for older adults care scenarios.

#### 3.2.2 Self-collected experimental setup

The experimental design of the self-collected dataset is consistent with the benchmark dataset experiments to ensure comparable results. For feature extraction, a time-series convolutional neural network (TS-CNN) was used to capture temporal patterns for time series of physiological and behavioral data (e.g., heart rate and activity level), while a clinical feature embedding (CFE) was used to generate low-dimensional representations for structured environmental parameters and clinical logs. In hypergraph construction, a multimodal hypergraph was constructed using KNN method (K = 5), where nodes represent individual participants and hyperedges capture higher-order relationships between physiological, behavioral, and environmental modalities.

The HGCN model configuration includes 2 layers of feature propagation and 2 fully connected layers for classification, with a hidden layer dimension of 128. A dropout rate of 0.5 was set to prevent overfitting, and the model was trained for 600 cycles using the Adam optimizer, with a learning rate of 0.001. the loss function uses cross-entropy, with a regularized weight decay of 0.0005. since disease risk prediction is a multiclassification task, the evaluation metrics accuracy and F1 score are selected. To fully evaluate performance, HGCN was compared with baseline models, including standard graph convolutional networks (GCN) and GraphSAGE, both of which were adapted to fit the hypergraph structure.

#### 3.2.3 Self-collected results and discussion

The results of the experiments on the self-collected dataset are shown in Table 5, which lists the accuracy and F1 scores of the HGCN and the baseline model. The HGCN achieved an accuracy of 87.26% and an F1 score of 0.831 on the disease risk prediction task, which is significantly better than literature (22) (accuracy of 82.33%, F1 score of 0.795), literature (23) (accuracy of 83.75%, F1 score of 0.806), literature (24) (accuracy of 84.12%, F1 score of 0.811) and literature (25) (accuracy of 84.45%, F1 score of 0.814). The results validates our method’s superiority. Additionally, paired t-tests confirm that our method significantly outperforms all baselines (*p* < 0.05).

**Table 5:** Performance comparison on self-collected dataset.

Method	Accuracy (%)	F1 score
Literature (22)	82.33	0.795
Literature (23)	83.75	0.806
Literature (24)	84.12	0.811
Literature (25)	84.45	0.814
Proposed	87.26	0.831

This result suggests that HGCN is able to effectively capture complex higher-order relationships among multimodal health data, which is particularly important in geriatric healthcare scenarios because of the deep correlations among physiological, behavioral, and environmental factors.

### 3.3 Ablation studies

To evaluate the contribution of different data modalities and hypergraph components to the proposed HGCN model, we conducted comprehensive ablation studies on the self-collected dataset. Two groups of experiments were designed:

We performed two sets of experiments. First, we assessed the model’s performance by excluding one data modality at a time while keeping the others intact. Specifically, we removed the physiological, behavioral, and environmental data separately and measured the resulting accuracy. Second, to examine the impact of the adaptive weighting mechanism, we replaced it with a static uniform weighting scheme in the full model and compared the performance. The results of these experiments are summarized in Table 6.

**Table 6:** Ablation study results.

Ablation setting	Accuracy (%)
Full model (all modalities, adaptive weights)	87.26
Without physiological data	78.45
Without behavioral data	80.12
Without environmental data	82.67
Static uniform weighting (vs. adaptive)	84.33

The results show that excluding physiological data resulted in the largest accuracy decline (8.81%), indicating that physiological signals (e.g., heart rate, blood pressure) are the most critical modality for disease risk prediction in older adults healthcare scenarios. Removal of behavioral data (e.g., activity levels, sleep patterns) also led to a notable performance drop (7.14%), highlighting its complementary role. Environmental data contributed to a lesser extent (4.59% decrease), but its inclusion still enhanced prediction accuracy, demonstrating the value of incorporating ambient contextual factors. Moreover, replacing the adaptive weighting mechanism with static uniform weights resulted in a 2.93% accuracy decrease, confirming that dynamic weighting enables the model to emphasize more informative features and temporal segments effectively, thereby improving predictive performance.

## 4 Conclusion

This study proposes an intelligent healthcare management framework for the older adults population based on HGCN, aiming to address the challenges posed by population aging to the traditional healthcare system. The framework integrates multimodal health data through wearable devices and IoT sensors, constructs a dynamic medical knowledge graph, and combines hypergraph convolution with rooted hierarchical feature learning algorithms to achieve accurate analysis of health data and collaborative management across organizations. Experiments on UCI and PhysioNet benchmark datasets show that the framework outperforms existing graph methods and hypergraph methods in classification tasks. In addition, experiments on a self-collected dataset further validate the applicability of the framework in real healthcare scenarios, achieving significant results in disease risk prediction. System performance tests confirm the stability and reliability of the platform under high load, making it a solid solution for integrated healthcare services. This study provides a novel approach to enhance the efficiency and quality of geriatric healthcare services, optimize the allocation of healthcare resources, and provide personalized and intelligent health management.

While the proposed framework demonstrates promising results, several limitations warrant attention. First, the self-collected dataset used in our experiments, although valuable for proof of concept, is relatively small, comprising only 50 participants. This limited sample size may affect the generalizability of our findings to broader populations or different healthcare settings. Second, the study does not extensively address the interpretability of model predictions, which is critical for clinical decision-making where transparent reasoning processes are required to build trust and ensure safe implementation. Future work will prioritize: (1) Expanding the dataset to include a larger and more diverse population to enhance the robustness and generalizability of the model; (2) Enhancing the interpretability of model outputs by integrating explainable AI techniques that provide clinicians with clear, understandable rationales for each prediction, thereby supporting informed and transparent clinical decisions. Furthermore, data security and privacy protection measures will be strengthened to ensure compliance with HIPAA and GDPR regulations, including implementing end-to-end encryption, robust access controls, and privacy-preserving computation techniques to safeguard sensitive health information.

## Data Availability

The original contributions presented in the study are included in the article/supplementary material, further inquiries can be directed to the corresponding author.
